# A multi-layer regulatory landscape of chilling requirement revealed by stage-resolved transcriptomics and hormone profiling in peach floral buds

**DOI:** 10.3389/fpls.2026.1819363

**Published:** 2026-04-24

**Authors:** Weihan Zhang, Haiyan Li, Yayun Sun, Yuepeng Han, Baoxiong Wan, Liao Liao

**Affiliations:** 1 Guangxi Key Laboratory of Germplasm Innovation and Utilization of Specialty Commercial Crops in North Guangxi, Guangxi Academy of Specialty Crops, Guilin, Guangxi, China; 2 State Key Laboratory of Plant Diversity and Specialty Crops, Wuhan Botanical Garden of Chinese Academy of Sciences, Wuhan, China; 3 University of Chinese Academy of Sciences, Beijing, China; 4 Sino-African Joint Research Center, Chinese Academy of Sciences, Wuhan, China

**Keywords:** chilling requirement, dormancy, hormone, peach, transcriptomics

## Abstract

Bud dormancy and chilling requirement (CR) are fundamental to the climate adaptation of temperate fruit trees, yet the regulatory mechanisms translating winter chill into genotype-specific dormancy release remain poorly resolved. Here, we generated a stage-resolved transcriptomic and hormone dataset from peach floral buds of four cultivars spanning contrasting CR phenotypes, sampled across endodormancy induction (S1), maintenance (S2), and release (S3), with matched ABA/GA measurements. By applying trajectory-centric analysis that standardizes temporal profiles within each cultivar, we partitioned dormancy progression into eight distinct transcriptional modules and identified those exhibiting CR-associated divergence. Candidate-restricted WGCNA resolved these CR-enriched genes into co-expression modules, with one module showing a significant positive correlation with ABA abundance. A cross-cultivar consensus network further nominated hub-centered regulatory candidates. Through integrated evidence prioritization that combined temporal divergence, network topology, cross-cultivar support, and functional annotation, we established a ranked and functionally diverse set of CR-associated candidate regulators, including transcription factors, kinase-related components, hormone-associated genes, and chromatin/RNA factors. Together, these analyses reveal a multi-layer regulatory landscape of CR in which divergence is reflected by stage-dependent remodeling of specific transcriptional and hormone-associated programs rather than a global transcriptome shift. This study provides a structured candidate resource for downstream mechanistic dissection and climate-resilient breeding in peach and related woody perennials.

## 1 Introduction

Bud dormancy is a defining adaptive strategy of temperate woody perennials, enabling overwinter survival and ensuring that reproductive development resumes only when spring conditions are favorable (Lang et al., 1987; Rohde and Bhalerao, 2007; Sabir et al., 2025). In deciduous fruit trees, endodormancy release requires the accumulation of a cultivar-specific chilling requirement (CR) (Zhang et al., 2024a), after which buds regain growth competence and subsequently progress under heat accumulation to reach budburst and bloom (Coville, 1920; Cooke et al., 2012). Under climate change, many major production areas are experiencing warmer and more variable winters, increasing the frequency of insufficient chilling, asynchronous flowering, and unstable yields (Campoy et al., 2011; Luedeling, 2012; Guillamón et al., 2025). These phenological disruptions are not only agronomically damaging but also complicate forecasting and orchard management because “effective chill” depends on temperature exposure patterns and can be partly negated by warm spells in some chill models (Richardson et al., 1974; Fishman et al., 1987). As a result, CR has become a central trait for climate-resilient breeding and cultivar deployment, yet the mechanisms that translate winter temperature history into stable, genotype-specific dormancy release remain incompletely resolved (Cooke et al., 2012; Ding et al., 2024).

A practical reason why CR remains difficult to model and breed is that its phenotyping and interpretation are intrinsically context-dependent. Classical chill models (e.g., Chilling Hours, Utah model/Chill Units, and Dynamic model) implement different assumptions about temperature effectiveness and the negation of chill by warm episodes, which can lead to divergent CR estimates for the same genotype across environments (Weinberger, 1950; Richardson et al., 1974). Physiological experiments further indicate that dormancy depth changes gradually over time and that similar “chill totals” can be achieved through different thermal histories with different biological outcomes, particularly under warm-winter regimes (Erez and Lavee, 1971; Hänninen, 2016). At the molecular level, dormancy transition requires coordinated remodeling across multiple regulatory layers—including hormone balance and sensitivity, transcriptional control, chromatin state, and post-transcriptional regulation, suggesting that CR is unlikely to be explained by a single pathway alone (Horvath et al., 2003; Liu and Sherif, 2019; Zhang et al., 2024a). Moreover, transcriptome-wide comparisons among cultivars can be dominated by genotype background effects, potentially masking CR-associated signals unless analyses explicitly focus on stage-resolved dynamics rather than baseline differences (Cooke et al., 2012; Zhang et al., 2024b).

Peach (Prunus persica) is uniquely valuable for dissecting CR because it is simultaneously an economically important crop exposed to winter warming and a genetically tractable “model fruit tree” with rich genomic resources (Guillamón et al., 2025). Peach shows broad natural and breeding-derived variation in CR and bloom phenology, enabling mechanistic interrogation of how winter chill translates into reproductive timing differences among cultivars (Weinberger, 1950; Fan et al., 2010). From a genetic standpoint, peach is diploid with a high-quality reference genome and extensive mapping resources, providing a robust framework for linking phenological traits to genes and regulatory networks (Verde et al., 2013; Zhebentyayeva et al., 2014; Yang et al., 2024). Peach also offered a landmark dormancy mutant (evergrowing/evergreen), which enabled positional and functional candidate discovery at the evergrowing (EVG) locus (Rodriguez-A. et al., 1994; Bielenberg et al., 2008). Work stemming from EVG established a tandem cluster of dormancy-associated MADS-box genes (DAMs) as central regulators of dormancy-related growth cessation and bud formation, and seasonal DAM expression dynamics have since become a reference point across Prunus dormancy studies (Li et al., 2009; Yamane et al., 2019). Critically, peach also provides a strong mechanistic bridge between genotype and molecular regulation, as cultivar-dependent chromatin regulation at DAM loci and functional evidence for PpDAM6 in CR-mediated dormancy release have now been demonstrated (Leida et al., 2012; Canton et al., 2022; Zhao et al., 2023).

Even with these advances, several gaps limit a transferable understanding of CR that can be generalized beyond a handful of “known genes.” Genetic mapping and association studies highlight both major-effect and polygenic contributions to CR and bloom date, but mapping resolution and allelic heterogeneity can obscure how loci act across developmental time and across genetic backgrounds (Romeu et al., 2014; Bielenberg et al., 2015). Mechanistically, accumulating evidence indicates that DAM/SVP-centered repression intersects with hormone pathways—particularly ABA–GA antagonism—and is embedded within broader remodeling of signaling responsiveness, transcriptional competence, and chromatin state (Tuan et al., 2017; Liu and Sherif, 2019; Hsiang et al., 2024). Meanwhile, seasonal timing pathways (including circadian/photoperiodic control) can gate responsiveness to temperature and hormones, potentially shaping when CR-associated divergence becomes detectable during dormancy progression (Böhlenius et al., 2006; Singh et al., 2020). Finally, many transcriptomic studies contrast cultivars at one or two time points, which can miss stage windows where CR divergence is maximal and can confound CR with background genotype effects, limiting candidate prioritization for downstream community use (Ding et al., 2024).

Here, we leverage peach as both a high-impact crop and an informative genetic system to build a stage-resolved, background-aware framework for CR regulation in flower buds. We profile four cultivars spanning contrasting CR phenotypes across endodormancy entry, maintenance, and release, together with matched ABA/GA measurements to anchor transcriptional programs in physiological state. We then apply a trajectory-centric strategy that standardizes temporal profiles within each cultivar to prioritize the direction and timing of dormancy programs rather than baseline cultivar differences, enabling identification of CR-divergent temporal modules. Next, we elevate CR-informative programs into a candidate-restricted co-expression framework and cross-cultivar consensus network to nominate hub/bridge candidates embedded within coherent regulatory subsystems. Finally, we provide an evidence-ranked, functionally diverse candidate resource for the community, offering a structured foundation for future mechanistic dissection and climate-resilient breeding of chilling adaptation in peach and related woody perennials.

## 2 Materials and methods

### 2.1 Plant materials and chilling requirement evaluation

Floral buds of four peach (*Prunus persica*) cultivars with contrasting chilling requirement (CR) phenotypes were collected from an experimental orchard located in Wuhan Botanical Garden, Wuhan, Hubei Province, China. The selected genotypes included two low-CR cultivars (‘NG’ and ‘NS’) and two high-CR cultivars (‘CM’ and ‘ZY’). The chilling requirement for each cultivar was estimated using the 7°C model. Sampling was systematically conducted at three critical developmental stages representing the progression of endodormancy: induction (S1), maintenance (S2), and release (S3). At each designated time point, floral buds were excised from healthy shoots of uniform vigor, immediately snap-frozen in liquid nitrogen, and stored at -80 °C until subsequent RNA extraction and hormone quantification. Each biological condition consisted of 3 independent biological replicates.

### 2.2 Endogenous hormone extraction and quantification

Endogenous phytohormones were extracted and quantified using an UPLC-ESI-MS/MS system. High-purity standards were obtained from Olchemim Ltd. (Czech Republic) and isoReag (China), with HPLC-grade reagents sourced from Merck and Sigma-Aldrich. Briefly, 50 mg of homogenized floral bud powder was extracted with 1 mL of a methanol/water/formic acid mixture (15:4:1, v/v/v) supplemented with 10 μL of internal standard (100 ng/mL). After 10 min of vortexing and 5 min of centrifugation (12,000 rpm at 4 °C), the supernatant was evaporated to dryness, reconstituted in 100 μL of 80% methanol, and filtered through a 0.22 μm membrane. The extracts were analyzed using an ExionLC™ AD UPLC system coupled with a QTRAP^®^ 6500+ mass spectrometer (Sciex). Chromatographic separation was achieved on a Waters ACQUITY UPLC HSS T3 C18 column (100 mm × 2.1 mm, 1.8 μm) at 40 °C, using a mobile phase of water (A) and acetonitrile (B), both containing 0.04% acetic acid, with a flow rate of 0.35 mL/min. The ESI source operated in both positive and negative ion modes (550 °C, 5500 V/-4500 V) using scheduled multiple reaction monitoring (MRM). Data acquisition and metabolite quantification were performed using Analyst 1.6.3 and Multiquant 3.0.3 software (Sciex), with individual MRM transitions optimized for declustering potentials (DP) and collision energies (CE) (Pan et al., 2010; Šimura et al., 2018).

### 2.3 RNA extraction, library construction, and transcriptomic data processing

Total RNA was isolated from the floral buds utilizing the MGIEasy RNA Extraction Kit (MGI, Wuhan, China), according to the manufacturer’s protocol. RNA integrity and purity were rigorously evaluated using a NanoDrop spectrophotometer and an Agilent 2100 Bioanalyzer. Sequencing libraries were constructed and sequenced on an MGISEQ-T7 platform to generate paired-end reads. The raw sequencing data were processed using fastp (Chen et al., 2018) software to trim adapter sequences and filter out low-quality reads. The resulting high-quality clean reads were then aligned to the *Prunus persica* reference genome (v2.0.a1) using the HISAT2 aligner (Kim et al., 2019). Gene expression levels were quantified and normalized as Transcripts Per Kilobase Million (TPM) using StringTie (Pertea et al., 2015). To assess biological reproducibility and global sample variance, Principal Component Analysis (PCA) was executed based on the TPM expression matrix.

### 2.4 Trajectory-centric clustering and functional enrichment analysis

To delineate the stage-resolved transcriptional programs governing dormancy transitions, we implemented a trajectory-centric analytical framework. Initially, the mean TPM values for each expressed gene were calculated across the S1–S3 stages within each individual cultivar. To mitigate baseline expression biases and focus on temporal dynamics, these expression trajectories were z-score standardized intra-cultivar. The standardized profiles were subsequently partitioned into eight discrete transcriptional modules (C1–C8) utilizing the *k*-means clustering algorithm (*k* = 8). Clusters exhibiting significant transcriptional divergence between the low-CR and high-CR groups were selected for further interrogation. Functional enrichment analysis for Gene Ontology (GO), InterPro (IPR) domains, and KEGG pathways within each identified module was performed using hypergeometric tests, with the significance threshold rigorously set at an adjusted False Discovery Rate (FDR) < 0.05.

### 2.5 Co-expression network modeling and candidate prioritization

For the CR-informative transcriptional modules (C1–C6), a multi-layered network analysis was conducted to pinpoint candidate regulatory elements. To uncouple genotypic variations from predominant developmental stage effects, the expression matrix was first residualized by stage. Pearson correlation coefficients were computed on the stage-residualized data to infer co-expression interactions, and intra-module hub genes were identified based on node degree connectivity. Furthermore, Weighted Gene Co-expression Network Analysis (WGCNA) was applied to the prioritized gene pool. Linear models (Module Eigengene ~ Stage + Cultivar + CR_group + Hormones) were fitted to evaluate module-trait associations rigorously. To ensure the cross-genotype consistency of the regulatory network, a consensus network was constructed by retaining exclusively those edges that exhibited consistent correlation directions in at least three (e.g., three out of four) cultivars. The finalized consensus network was exported and visualized using Cytoscape software (Shannon et al., 2003). Ultimately, candidate regulators for qPCR validation were prioritized based on network topology (hub status), CR-associated expression divergence, and a basal transcript abundance threshold (mean TPM ≥ 0.5 and maximum TPM ≥ 1).

## 3 Results

### 3.1 Global transcriptome quality, dormancy-stage progression, and hormone trajectories define the molecular landscape of CR-divergent peach floral buds

**Figure 1:**
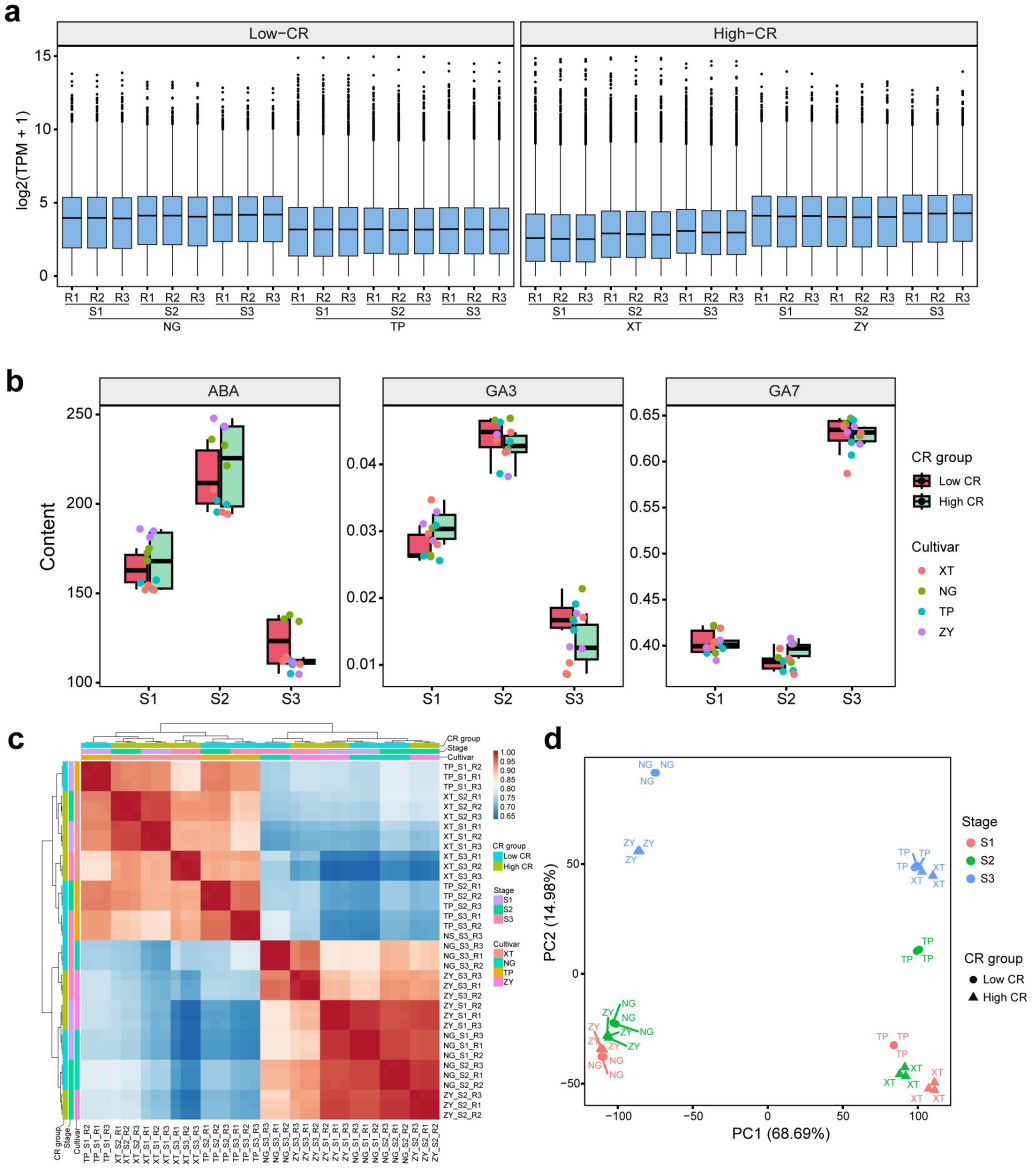
Global transcriptome quality, dormancy-stage structure, and hormone trajectories in CR-divergent peach floral buds. **(a)** Distribution of gene expression values across all 36 libraries shown as boxplots of log2(TPM + 1) for each sample. Samples are ordered by cultivar, dormancy stage (S1–S3), and biological replicate, illustrating highly comparable expression distributions with no obvious outliers or global shifts indicative of major technical artifacts. Side facets indicate CR groups (Low-CR vs High-CR). **(b)** Hormone dynamics across dormancy stages. Boxplots show the abundance of ABA, GA3, and GA7 measured in the same floral-bud samples, stratified by dormancy stage and CR group, with individual points representing biological replicates and colors indicating cultivars. ABA peaks at S2 and decreases at S3, while GA7 exhibits a marked increase at S3, providing a physiological context for stage-dependent transcriptional reprogramming. **(c)** Sample-to-sample similarity assessed by Pearson correlation of log2(TPM + 1) expression profiles (genes filtered for robust expression). The correlation heatmap and hierarchical clustering show tight grouping of biological replicates within each cultivar and stage, supporting high reproducibility and low technical variation. Annotation bars indicate cultivar, dormancy stage, and CR group. **(d)** Principal component analysis (PCA) of samples based on the most variable genes. Each point represents one library, colored by dormancy stage and shaped by CR group; cultivar labels are overlaid. PC1 primarily captures cultivar background effects, whereas PC2 aligns with dormancy progression from S1 to S3, indicating coherent stage-resolved transcriptome dynamics.

We established a stage-resolved transcriptomic and hormonal dataset to investigate chilling requirement (CR) divergence in peach floral buds by profiling four cultivars with contrasting CR phenotypes (Low-CR: NG and NS; High-CR: CM and ZY) across three endodormancy stages (S1, dormancy induction; S2, dormancy maintenance; S3, dormancy release) (Weinberger, 1950), with three independent biological replicates per cultivar–stage combination (36 libraries). Transcript abundance was quantified as TPM at the genome scale, yielding expression estimates for 26,873 annotated genes; for exploratory analyses and quality assessment, we retained genes with TPM ≥ 1 in at least three of the 36 libraries, resulting in 21,931 expressed genes used in downstream QC and multivariate analyses. Across the 36 libraries, log2(TPM + 1) distributions were highly consistent, showing comparable central tendencies and dispersion without pronounced sample-wide shifts, supporting the absence of major library-quality artifacts, and providing an initial indication that the dataset is suitable for sensitive comparisons (**Figure 1A**). Tight replicate concordance was further supported by sample-to-sample Pearson correlation analysis, in which biological replicates clustered closely within each cultivar and dormancy stage, with mean within-group correlations ranging from 0.990 to 0.998 (**Figure 1C**). These QC results indicate that technical variability is low relative to biological structure, enabling robust inference of stage-dependent programs and phenotype-associated signals.

To characterize the dominant sources of transcriptional variation, we performed principal component analysis (PCA) using the most variable genes. The first two components captured the majority of variance (PC1, 68.69%; PC2, 14.98%), indicating that a limited set of macro-level factors shapes the expression landscape. Notably, PC1 primarily separated samples by cultivar identity, underscoring strong genotype effects typical of woody perennials (Niu et al., 2024). CR group is confounded with cultivar identity (each cultivar belongs to one CR group), therefore PC1 separation also aligns with CR group to some extent. In contrast, PC2 aligned closely with dormancy progression, with samples displaying an ordered displacement from S1 to S2 to S3 (**Figure 1D**). This stage-associated ordering suggests coherent transcriptomic reprogramming accompanying the endodormancy transition, while the dominance of cultivar background along PC1 implies that CR-associated differences are unlikely to appear as a uniform transcriptome-wide shift across all samples and stages. Instead, CR-linked signals are more plausibly embedded within specific stage-dependent subprograms that become detectable when expression dynamics are examined in a stratified or modular manner.

Hormone measurements obtained from the same samples provided an orthogonal physiological context for interpreting transcriptome dynamics. Abscisic acid (ABA) exhibited a pronounced stage dependence, increasing from S1 to S2 and declining at S3 (Tuan et al., 2017), consistent with ABA-associated maintenance of dormancy and its reduction during release; GA_3_ showed stage-dependent variation, whereas GA_7_ displayed a distinct pattern with a marked elevation at S3 relative to earlier stages (**Figure 1B**). Together, the high reproducibility of transcript abundance, clear stage-resolved structure, and coordinated ABA/GA trajectories establish a robust foundation for linking CR divergence to stage-specific transcriptional programs and hormone-associated regulatory modules.

### 3.2 Trajectory clustering reveals stage-resolved transcriptional modules associated with chilling requirement

**Figure 2:**
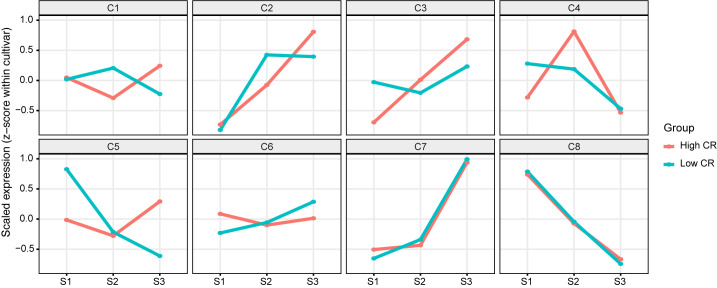
Trajectory clustering reveals CR-associated transcriptional programs across dormancy stages. Genes expressed in floral buds were clustered by their standardized temporal profiles across S1–S3 (within-cultivar z-score scaling), yielding eight trajectory clusters (C1–C8). Lines represent cluster-average trajectories for Low-CR and High-CR groups, highlighting clusters with conserved stage trends as well as clusters showing CR-dependent amplitude/timing shifts.

Trajectory-centric analysis was used to partition dormancy progression into interpretable temporal programs while minimizing baseline differences among cultivars. After filtering for robust expression, 21,931 genes were retained for trajectory modeling, and functional annotation coverage among expressed genes was substantial (13,653 GO-mapped genes; 17,716 InterPro-mapped genes; 6,858 KO-mapped genes; 4,424 KEGG-pathway–mapped genes), enabling multi-layer interpretation of cluster composition. Expression trajectories were standardized within each cultivar (z-score across S1–S3) and clustered by k-means (k = 8), yielding eight recurrent modules (C1–C8) that capture distinct temporal patterns across endodormancy establishment (S1), maintenance (S2), and release (S3) (**Figure 2**). Because clustering was performed on within-cultivar standardized profiles, the modules primarily reflect stage-dependent dynamics (direction and timing of change) rather than cultivar-dependent baselines; accordingly, the Low-CR and High-CR group means are broadly concordant for several modules, while other modules show visibly different amplitudes or temporal profiles between groups (**Figure 2**), indicating that CR-associated differences are concentrated in a subset of temporal programs rather than uniformly distributed across all dormancy-responsive genes.

Functional enrichment analyses supported the biological coherence of these modules and provided complementary mechanistic context at multiple annotation layers. At the GO level (**Supplementary Figure 1**), clusters were associated with recurrent themes spanning translation/ribosome functions, protein phosphorylation/kinase activity, and DNA-binding/transcriptional regulation, suggesting coordinated regulation of core cellular capacity, signaling, and gene regulatory control during dormancy transitions. InterPro domain enrichment (**Supplementary Figure 2**) reinforced these themes at the protein-feature level, with modules enriched for protein kinase(-like) domains/active sites and multiple regulatory domain families, consistent with signaling and transcriptional control being embedded within specific temporal trajectories. KO enrichment (**Supplementary Figure 3**) provided conserved functional components aligned with these processes (e.g., regulatory proteins and transport-associated components), while KEGG pathway enrichment (**Supplementary Figure 4**) further elevated cluster interpretation to pathway-scale programs. Notably, pathway enrichments included ribosome-related pathways (Ribosome; Ribosome biogenesis in eukaryotes) (Lyu et al., 2025), as well as multiple information-processing pathways such as Spliceosome/RNA transport/Proteasome, consistent with broad remodeling of translational and RNA–protein homeostasis across dormancy stages. In addition, metabolism- and physiology-associated pathways were recovered in a cluster-specific manner (e.g., pathways linked to carbohydrate metabolism and photosynthesis in the dotplot), and regulatory physiology pathways such as Plant hormone signal transduction and Circadian rhythm – plant were also identified (Zhao et al., 2025) (**Supplementary Figure 4**). Together, these analyses establish that the trajectory clusters correspond to coherent biological modules and provide a structured basis for prioritizing CR-relevant gene sets for downstream candidate nomination and network-level analyses, without requiring assumptions about a uniform stage trend across all modules.

### 3.3 CR-divergent trajectory modules nominate hub-centered regulatory candidates for downstream validation

**Figure 3:**
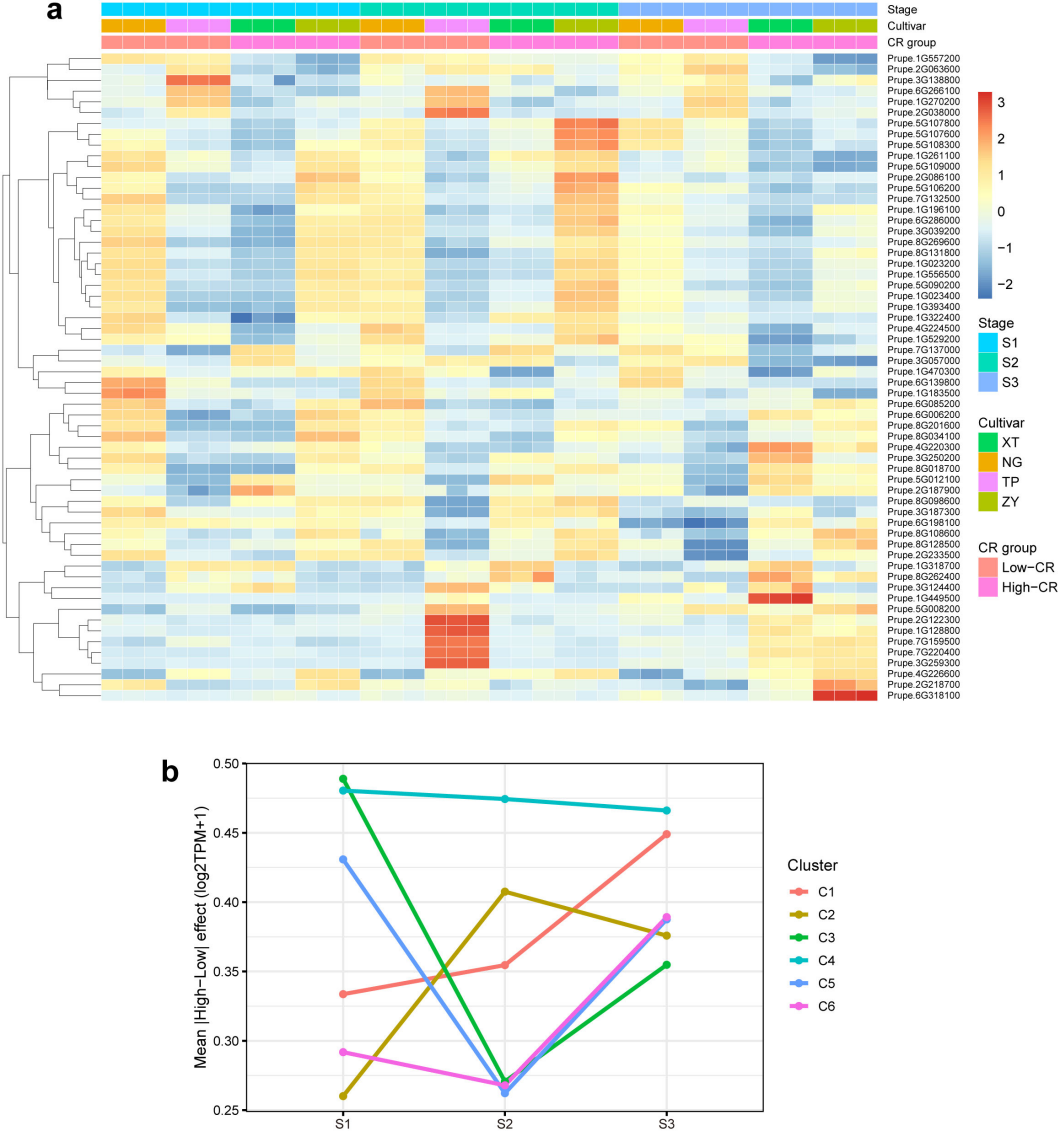
CR-divergent trajectory modules and prioritized candidate genes across dormancy stages **(a)** Heatmap showing the expression patterns of prioritized candidate genes from trajectory modules C1–C6 across all samples. Values represent row-scaled (z-score) log2(TPM+1) expression for each gene, enabling comparison of relative dynamics across stages; columns are ordered by Stage (S1-S3) and grouped by CR group (Low-CR vs High-CR), with additional sample annotations (cultivar/stage/CR group) shown as top bars. This panel visualizes that the prioritized set exhibits coherent stage structure and module-specific expression programs, with CR-associated differences concentrated in specific stage blocks rather than appearing as a uniform shift across the entire transcriptome. **(b)** Module-level summary of CR-associated divergence for trajectory clusters C1–C6 across stages. For each cluster and stage, divergence is quantified as the mean absolute High-CR minus Low-CR difference in log2(TPM+1), calculated on cultivar-stage replicate means (i.e., treating cultivars as the independent units). Lines connect S1–S3 to highlight that the magnitude of divergence is both module-dependent and stage-dependent, revealing distinct “windows” of maximal separation among clusters during dormancy progression.

Having defined eight recurrent trajectory modules that capture stage-dependent dormancy programs, we next asked which modules preferentially harbor CR-associated transcriptional divergence during dormancy progression. Rather than treating all modules equivalently, we prioritized C1–C6 because these modules contained substantial fractions of genes exhibiting directional discordance between High-CR and Low-CR group means across S1–S3 (C1: 1,440/2,006; C2: 1,415/2,205; C3: 1,402/2,272; C4: 1,480/2,371; C5: 1,832/2,639; C6: 1,587/2,490), whereas C7–C8 displayed largely parallel group-mean trajectories and were not pursued further. Consistent with this prioritization, stage-resolved contrasts in log_2_(TPM + 1) showed that the magnitude of High–Low divergence is module- and stage-dependent (**Figure 3B**). Specifically, C3 and C5 exhibited their highest divergence at S1 (mean |High–Low| ≈ 0.49 and 0.43, respectively), C2 peaked at S2 (≈ 0.41), and C1 showed increasing divergence toward S3 (≈ 0.45). In contrast, C4 maintained a persistently elevated divergence across stages (≈ 0.48–0.47 from S1 to S3), indicating that CR-associated differences within this program are not confined to a single dormancy phase (Jin et al., 2026) (**Figure 3B**). Notably, these patterns do not imply a uniform “late-stage amplification” of divergence; instead, different temporal programs display distinct windows of maximal separation between CR groups.

Since CR group membership is not fully orthogonal to cultivar identity (i.e., each group comprises distinct cultivars), the High–Low contrasts are interpreted as CR-associated group differences rather than as definitive causal effects of CR per se. To mitigate over-interpretation driven by any single genotype, candidate nomination therefore integrated trajectory divergence with a cross-cultivar concordance filter (consistent directionality in ≥3 of 4 cultivars), and was subsequently restricted to genes with regulatory plausibility based on functional annotations. This yielded a focused set of 676 prioritized genes across C1–C6 (C1: 97; C2: 116; C3: 106; C4: 167; C5: 111; C6: 79), whose standardized expression patterns exhibited coherent stage structure and module specificity in a consolidated heatmap (**Figure 3A**). The heatmap is used here to visualize that the prioritized set captures stage-resolved dynamics with concentrated High–Low differences across modules, rather than to claim universal separation of individual samples by CR group. Within each prioritized module, we further extracted high-connectivity hub-like genes from the co-expression structure as candidate regulatory anchors. Hubs were not distributed randomly across biological themes; instead, they highlighted interpretable axes that align with dormancy regulation. For example, C1 contained a prominent hub annotated as LUX (Prupe.1G120500; degree 397), together with other highly connected regulators including AGL19 (Prupe.8G267300; degree 267), consistent with the involvement of transcriptional timing control within a CR-divergent trajectory. C5 featured hubs spanning hormone perception and temporal regulation, including GID1B (Prupe.3G070500; degree 258), GI (Prupe.4G247200; degree 148), and APRR7/PRR7 (Prupe.4G028000; degree 167), alongside signaling components such as ABI1 (Prupe.6G004900; degree 173) and GAT1 (Prupe.8G031700; degree 310). In parallel, the broader prioritized set retained multiple hormone- and growth-related candidates in a cluster-specific manner (e.g., GA2OX8 in C1; PYR1 in C2; GH3.1 and BZR1 in C4; SAUR71 in C5), together suggesting that CR-associated divergence is embedded in discrete temporal programs that intersect clock-linked transcriptional regulation and hormone signaling. Collectively, these analyses connect module-level divergence (**Figure 3B**) to a tractable, annotation-supported shortlist (**Figure 3A**) and nominate hub-centred candidates spanning C1–C6 for downstream testing, while explicitly constraining interpretation to CR-associated group differences under partial cultivar confounding.

### 3.4 Candidate-restricted WGCNA resolves ABA-associated co-expression structure and cross-cultivar consensus hubs within CR-divergent programs

**Figure 4:**
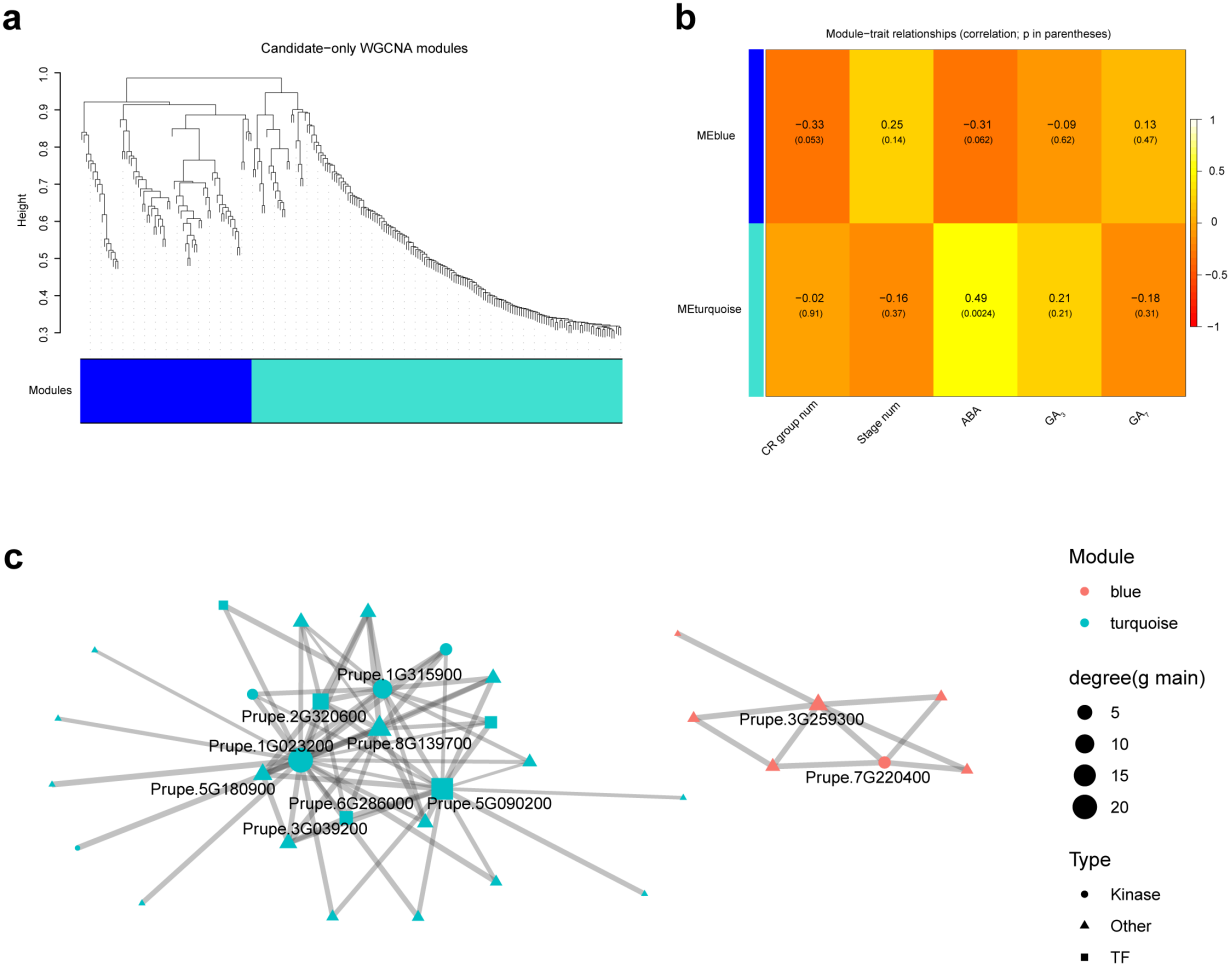
Candidate-restricted WGCNA resolves ABA-associated co-expression modules and a cross-cultivar consensus network within CR-divergent candidates. **(a)** Hierarchical clustering dendrogram of CR-divergent candidate genes (prioritized from trajectory-informed analyses in **Figure 3**) used for candidate-restricted WGCNA. The color bar below the dendrogram indicates module assignment, identifying two major co-expression modules (blue and turquoise) within the candidate space. **(b)** Module–trait relationship heatmap showing Pearson correlations between module eigengenes and sample traits (CR group num, Stage num, ABA, GA3, and GA7). Correlation coefficients are shown in each cell, with nominal p-values in parentheses. **(c)** Cross-cultivar consensus co-expression network derived from the key modules. Only connected genes (degree > 0) are displayed to emphasize the informative core topology. Node color indicates module membership (blue or turquoise), node size represents degree in the main graph, and node shape denotes functional category (TF, kinase, or other). Edge width reflects consensus-supported co-expression strength across cultivars. Labeled nodes highlight representative hub/bridge candidates within the network core.

Building on the CR-divergent candidate genes identified from trajectory-informed analyses, we next sought to organize these genes into a testable regulatory network structure by performing a candidate-restricted WGCNA. Because this analysis was intentionally confined to a pre-prioritized gene set (rather than the full transcriptome), the resulting modules should be interpreted as co-expression organization within CR-enriched candidates, not as an unbiased global transcriptome topology. The candidate-only WGCNA dendrogram resolved a small number of coherent co-expression modules, providing an explicit module-level framework for downstream trait association and network interpretation (**Figure 4A**). We then summarized each module by its eigengene and quantified module–trait relationships across dormancy-stage samples. This analysis identified two dominant modules with distinct biological associations: MEturquoise showed the strongest positive correlation with ABA abundance (r = 0.49, p = 0.0024), whereas MEblue displayed a modest but comparatively stronger trend with CR group (r = −0.33, p = 0.053), together with a weaker trend toward ABA association (r = −0.31, p = 0.062) (**Figure 4B**). These patterns suggest that CR-associated transcriptional divergence is not distributed uniformly across all candidate genes, but instead concentrates within specific co-expression modules, one of which is closely linked to ABA-related variation.

To further refine these modules into a biologically interpretable regulatory backbone, we constructed a cross-cultivar consensus co-expression network from the key modules, retaining only edges with concordant directionality in the majority of cultivars (≥3/4). This consensus filtering step was included specifically to reduce the possibility that apparent connectivity was driven by a single cultivar background. For visualization clarity, the main network panel displays only connected genes (degree > 0), thereby highlighting the informative core structure rather than isolated candidates (**Figure 4C**). The resulting network revealed a compact connected component enriched in signaling and regulatory factors, including kinase/phosphatase-associated genes, transcriptional regulators, and transport/homeostasis-related nodes, consistent with a coordinated signaling-to-transcription architecture within CR-divergent programs. In biological terms, the co-variation structure is consistent with an ABA-associated regulatory axis that integrates signaling responsiveness with downstream transcriptional and cellular reprogramming during dormancy progression, although the module-trait relationships and network edges are correlation-based and therefore define testable hypotheses rather than causal directionality. This framework provides a rigorous basis for final candidate prioritization and downstream functional interrogation.

### 3.5 Integrated evidence prioritization defines a candidate resource for chilling-requirement regulation

To refine the CR-associated transcriptomic landscape into a biologically interpretable and experimentally tractable set of regulators, we implemented an integrated evidence-prioritization framework that combines temporal divergence, network topology, cross-cultivar support, expression abundance, and functional annotation. Using the CR-divergent trajectory clusters identified above (C1–C6) together with the candidate-only network structure, each gene was assigned an evidence-based priority score to support downstream functional studies. Importantly, this ranking is intended to prioritize candidates for mechanistic investigation and does not, by itself, imply causal directionality.

**Figure 5:**
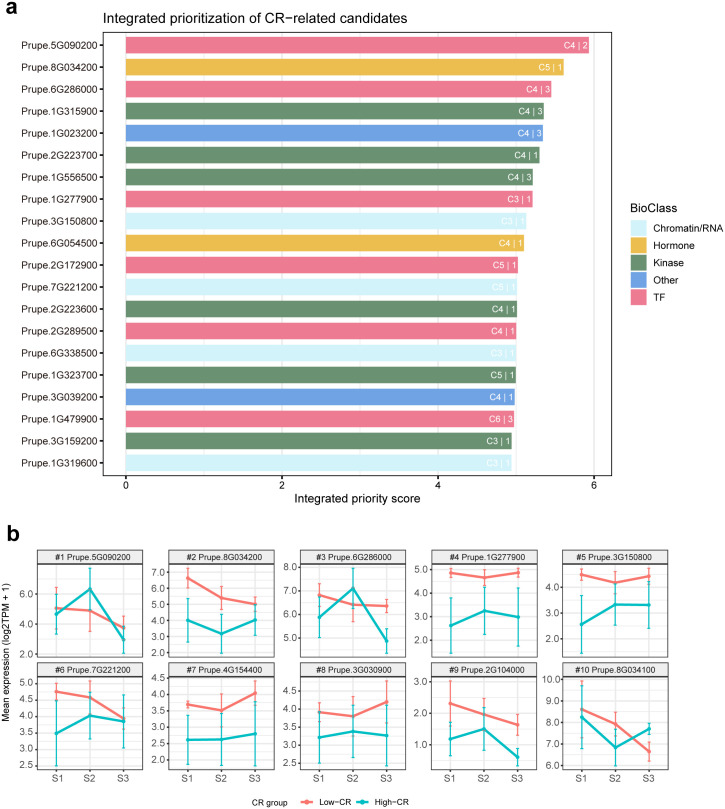
Integrated evidence prioritization defines a high-confidence candidate resource for chilling-requirement regulation. **(a)** Ranked prioritization of top CR-associated candidates based on an integrated evidence score that combines stage-resolved High-CR/Low-CR divergence, candidate-network topology (hub/bridge properties), cross-cultivar support, and expression abundance. Bar colors indicate functional class (TF, kinase-related, hormone-associated, chromatin/RNA-related, or other), and in-bar labels denote the trajectory cluster (C1–C6) and the dormancy stage at which the maximum CR-group divergence was observed (S1–S3). **(b)** Stage-resolved expression trajectories of the highest-priority candidates across dormancy progression (S1–S3), shown as group means for Low-CR and High-CR cultivars on the log2(TPM+1) scale with uncertainty bars indicating ± s.e.m. across the six libraries (2 cultivars × 3 biological replicates) within each CR group at each stage. Panels illustrate distinct modes of CR-associated regulation, including early-stage divergence, late-stage divergence, and stage-dependent reversals, supporting the view that CR-associated differences are distributed across multiple temporal programs rather than confined to a single stage.

Here, the integrated candidate universe was defined as all genes assigned to the CR-divergent trajectory clusters (C1–C6) after expression filtering. The 676-gene set described above was used only to construct the candidate-restricted network and to compute network-topology features (hub/bridge scores), which were then mapped back to the full C1–C6 universe for integrated scoring. Genes were eligible for ranking if they met the expression-abundance criterion (TPM ≥ 1 in ≥3 of the 36 libraries). From the final ranked list, we reported a focused panel of top candidates to preserve both strong evidence support and mechanistic diversity (**Figure 5A**).

The highest-ranked genes were supported by distinct but complementary lines of evidence. For example, Prupe.5G090200 (C4; TF) and Prupe.6G286000 (C4; TF) combined pronounced stage-resolved High-CR/Low-CR divergence with measurable centrality in the candidate-only network, consistent with roles in coordinated regulatory programs. In contrast, several other top-ranked genes (including Prupe.8G034200, Prupe.1G277900, Prupe.3G150800, and Prupe.7G221200) were prioritized primarily because they showed strong and reproducible CR-group separation across dormancy stages, even when they were not retained as highly connected nodes in the displayed main network (**Figure 5A**). This distinction is biologically important, as it indicates that informative CR-associated regulators can emerge either as network-organizing components or as robust stage-specific responders outside the densest connected subgraph.

Stage-resolved expression profiles of the top-ranked candidates further demonstrated that the prioritized genes capture multiple modes of CR-associated regulation rather than a single stereotyped pattern (**Figure 5B**). Some genes exhibited clear divergence in early dormancy (S1), whereas others showed stronger separation in late dormancy (S3) or stage-dependent reversals, suggesting that CR-related regulatory differences are distributed across the dormancy progression timeline. Notably, the shortlisted candidates also span multiple putative functional systems—including transcriptional control, signaling, hormone-associated regulation, and chromatin/RNA-associated processes—consistent with the multilayer organization inferred from trajectory clustering (**Figure 2**), candidate-level divergence analyses (**Figure 3**), and the candidate-only WGCNA framework (**Figure 4**). Collectively, these analyses define a prioritized, functionally diverse candidate resource for chilling-requirement regulation in peach flower buds. By integrating temporal behavior, network context, and cross-cultivar robustness, the resulting ranked list provides a structured foundation for future mechanistic studies, including molecular genetics, regulatory interaction assays, and comparative validation across germplasm panels.

## 4 Discussion

Dormancy is a dynamic developmental continuum in temperate perennials, and chilling requirement (CR) reflects how genotypes tune the timing and depth of this continuum to winter conditions. Our stage-resolved dataset—four cultivars spanning contrasting CR phenotypes profiled across three endodormancy stages with matched ABA/GA measurements—captures two core features that are often difficult to reconcile in tree systems: a strong cultivar (genetic background) effect and a coherent dormancy-stage progression. The dominance of cultivar identity along the first principal component, paired with orderly stage separation along the second, reinforces the broader concept that dormancy regulation is shaped by overlapping, genotype-dependent programs rather than a uniform transcriptome-wide switch (Cooke et al., 2012).

The paired hormone data provide an important physiological anchor for interpreting these temporal programs. The ABA rise from S1 to S2 and decline at S3, together with GA dynamics (including marked GA7 elevation at S3), is consistent with a widely supported framework in which ABA promotes dormancy maintenance whereas GA promotes growth competence and bud break, mediated through extensive hormonal cross-talk (Liu and Sherif, 2019). Our network-level results further suggest that this hormonal context is not merely correlative noise: in candidate-restricted WGCNA, one dominant module (MEturquoise) showed a clear positive association with ABA, implying that a substantial fraction of CR-divergent candidates co-varies with ABA-linked physiological state. While we do not infer causality from correlation-based networks, this convergence supports a working model in which ABA-associated signaling and downstream gene regulatory programs form a backbone of dormancy-stage remodeling. Upon this backbone, CR divergence may be superimposed through altered timing, amplitude, or gating of specific temporal modules.

Functional enrichment patterns across trajectory clusters and the candidate network reinforce this multi-layer interpretation. Recurrent enrichment for protein phosphorylation/kinase activity and transcriptional regulation highlights the likely importance of signal transduction and regulatory control during dormancy transitions, whereas enrichment for information-processing systems (e.g., RNA processing, proteostasis/translation-related themes) suggests that CR divergence may also be coupled to broader cellular capacity remodeling. This is consistent with the view that dormancy progression involves extensive metabolic and cellular reconfiguration, not only changes in a few “marker genes” (Cooke et al., 2012). Importantly, circadian- and hormone-related pathways also appeared among the enriched clusters. This pattern is notable in light of growing evidence that seasonal growth and dormancy are shaped by clock-linked gating of environmental responsiveness in trees (Singh et al., 2020). In this context, the hub-centered candidates nominated within CR-divergent modules provide concrete hypotheses for how clock–hormone and kinase–TF axes may intersect to shape CR-linked dormancy trajectories.

Our results naturally connect to established dormancy regulators in Prunus, particularly the dormancy-associated MADS-box (DAM) gene cluster identified at the peach evergrowing (evg) locus (Bielenberg et al., 2008; Li et al., 2009). Recent functional work demonstrating that PpDAM6 contributes to chilling-requirement–mediated dormancy and bud break further underscores the relevance of DAM-centered mechanisms for CR phenotypes (Zhao et al., 2023). Although our analysis was intentionally module-centric rather than anchored on known genes, the emergence of candidates annotated to signaling, transcriptional timing control, and hormone response suggests that CR divergence may be mediated by altered coupling among (i) DAM/SVP-like dormancy repression, (ii) ABA–GA balance and sensitivity, and (iii) clock-gated responsiveness that sets thresholds for progression toward release competence. This framing is consistent with the broader notion that ABA cross-talk and transcription factor networks modulate dormancy release in perennials (Pan et al., 2021).

Beyond mechanistic hypotheses, a practical contribution of this study is the generation of a structured, functionally diverse candidate resource for the community. By integrating trajectory-informed divergence, cross-cultivar concordance, and candidate-only network topology, we provide a ranked set of high-confidence CR-associated candidates that can be interrogated using diverse downstream approaches (genetic association, transient assays, stable transformation, gene editing, or comparative validation in broader germplasm panels). Candidate-restricted WGCNA itself is not intended to reconstruct the global transcriptome network, but rather to organize CR-enriched candidates into interpretable modules; this is in line with the intended use of co-expression networks as hypothesis-generating scaffolds (Langfelder and Horvath, 2008).

Several limitations motivate future work. First, CR group membership is partially confounded with cultivar identity in the current design, so High–Low contrasts should be interpreted as CR-associated group differences rather than isolated causal effects; expanding to larger panels where CR can be modeled as a continuous trait across many genotypes will improve identifiability and generalization. Second, three stages provide a clear backbone for dormancy progression but may miss finer transitions; denser time-series sampling (including ecodormancy) would better resolve phase boundaries and improve the temporal localization of CR divergence. Third, hormones were limited to ABA, GA3, and GA7; integrating additional hormones (ethylene, auxin, jasmonate, cytokinins), carbohydrates, and chromatin-level readouts (ATAC-seq or histone marks) would allow more explicit modeling of regulatory causality and network directionality. Finally, while consensus edge filtering reduces single-genotype-driven artifacts, correlation networks remain non-causal; integrating genetic variation (GWAS/QTL/SV) and perturbation experiments will be essential to validate the direction and hierarchy among nominated hubs, especially for candidates proposed to bridge signaling and transcription. Accordingly, the prioritized genes presented here should be viewed as inference-based candidates supported by multi-layered transcriptomic and physiological evidence, pending direct functional validation.

In summary, our study supports a model in which CR divergence is encoded as stage-dependent remodeling of specific temporal programs rather than a global transcriptome shift. By anchoring transcriptome dynamics to hormone trajectories and organizing CR-associated signals into trajectory clusters, co-expression modules, consensus hubs, and a ranked functional candidate resource, we provide both mechanistic hypotheses and a reusable analysis strategy for dissecting chilling requirement regulation in peach and related woody perennials (Cooke et al., 2012; Liu and Sherif, 2019; Zhao et al., 2023).

## Data Availability

The original contributions presented in the study are included in the article/**Supplementary Material**. Further inquiries can be directed to the corresponding authors.

## Supplementary material

Supplementary Figure 1 GO term enrichment for genes in each trajectory cluster (C1–C8).

Supplementary Figure 2 InterPro (IPR) domain enrichment for each trajectory cluster.

Supplementary Figure 3 KEGG ortholog (KO) enrichment for each trajectory cluster.

Supplementary Figure 4 KEGG pathway enrichment for each trajectory cluster.
