# Supplementary material for: A multi-layer regulatory landscape of chilling requirement revealed by stage-resolved transcriptomics and hormone profiling in peach floral buds
Source: Front Plant Sci. 2026 Apr 24;17:1819363. doi: 10.3389/fpls.2026.1819363 (PMC13152854; doi:10.3389/fpls.2026.1819363)
Supplement: Supplementary Figure 1 — GO term enrichment for genes in each trajectory cluster (C1–C8). [file Table1.docx]

Supplementary Material


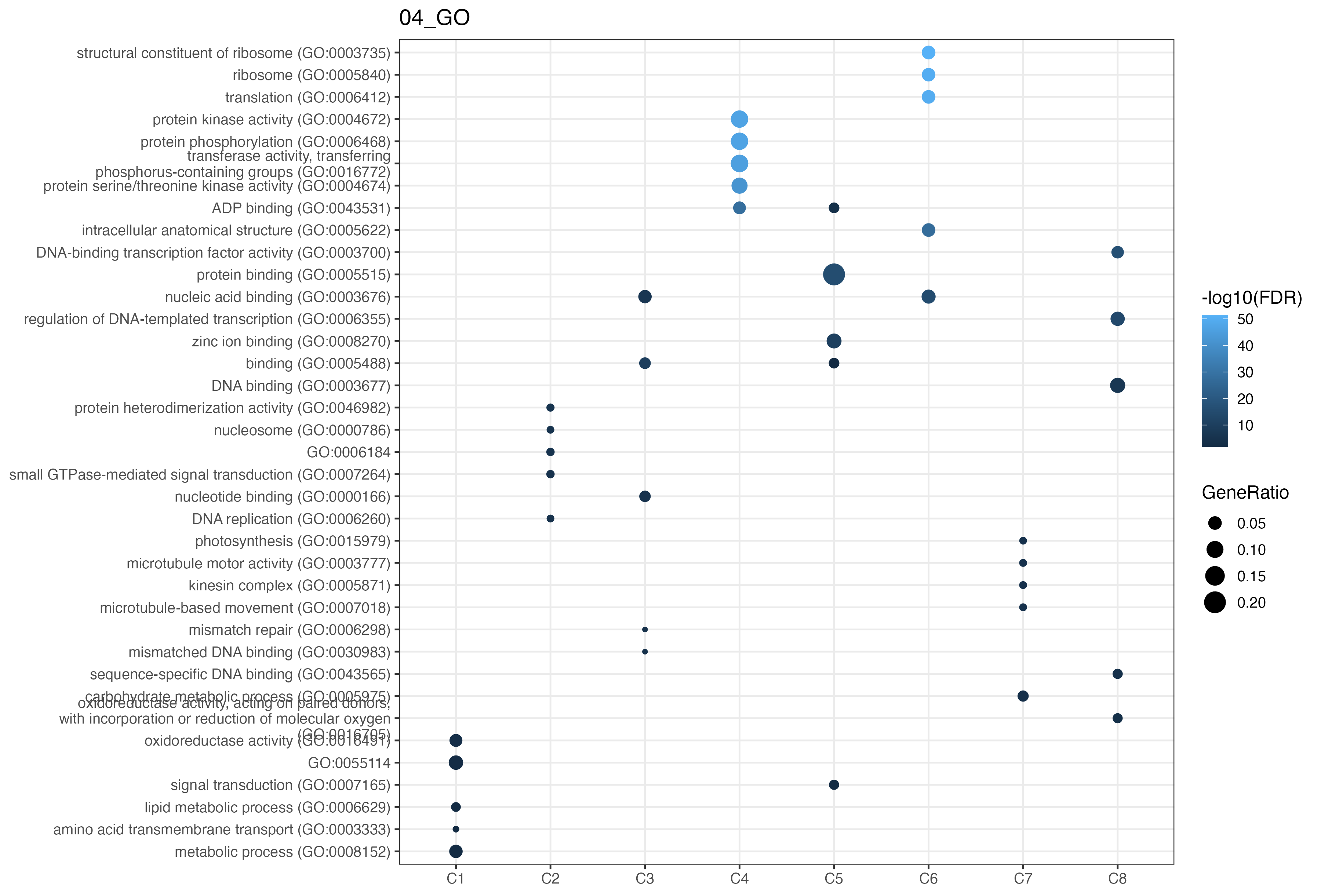


**Supplementary Figure 1.** **GO term enrichment for genes in each trajectory cluster (C1–C8).** Dot size indicates the gene ratio (proportion of cluster genes annotated to the term), and dot colour denotes enrichment significance (−log10 FDR) based on multiple-testing correction.


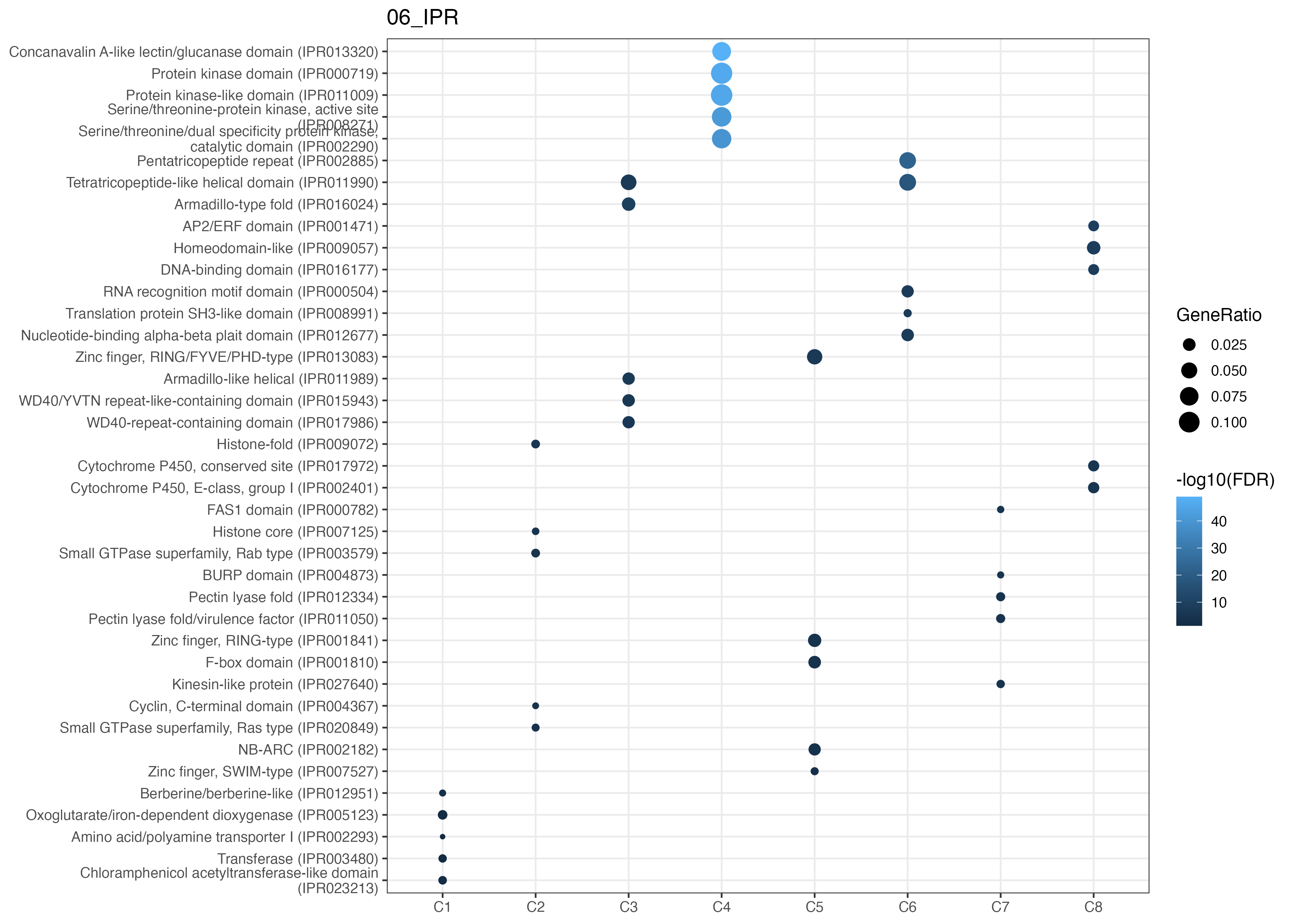


**Supplementary Figure 2. InterPro (IPR) domain enrichment for each trajectory cluster.** Enriched domains summarise over-represented protein functional architectures within clusters; dot size represents the gene ratio and dot colour indicates −log10(FDR).


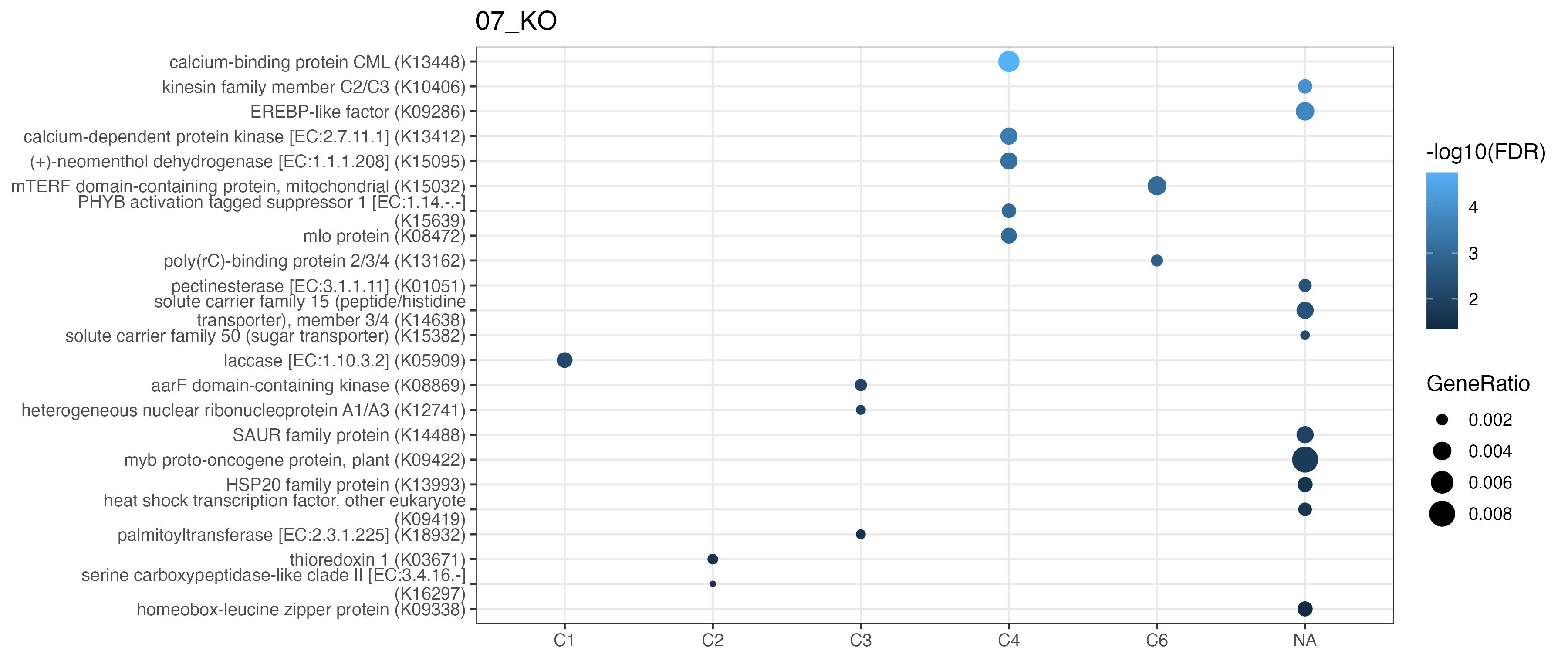


**Supplementary Figure 3. KEGG ortholog (KO) enrichment for each trajectory cluster.** KO-level enrichment highlights conserved functional components over-represented in cluster genes; dot size indicates gene ratio and dot colour indicates −log10(FDR).


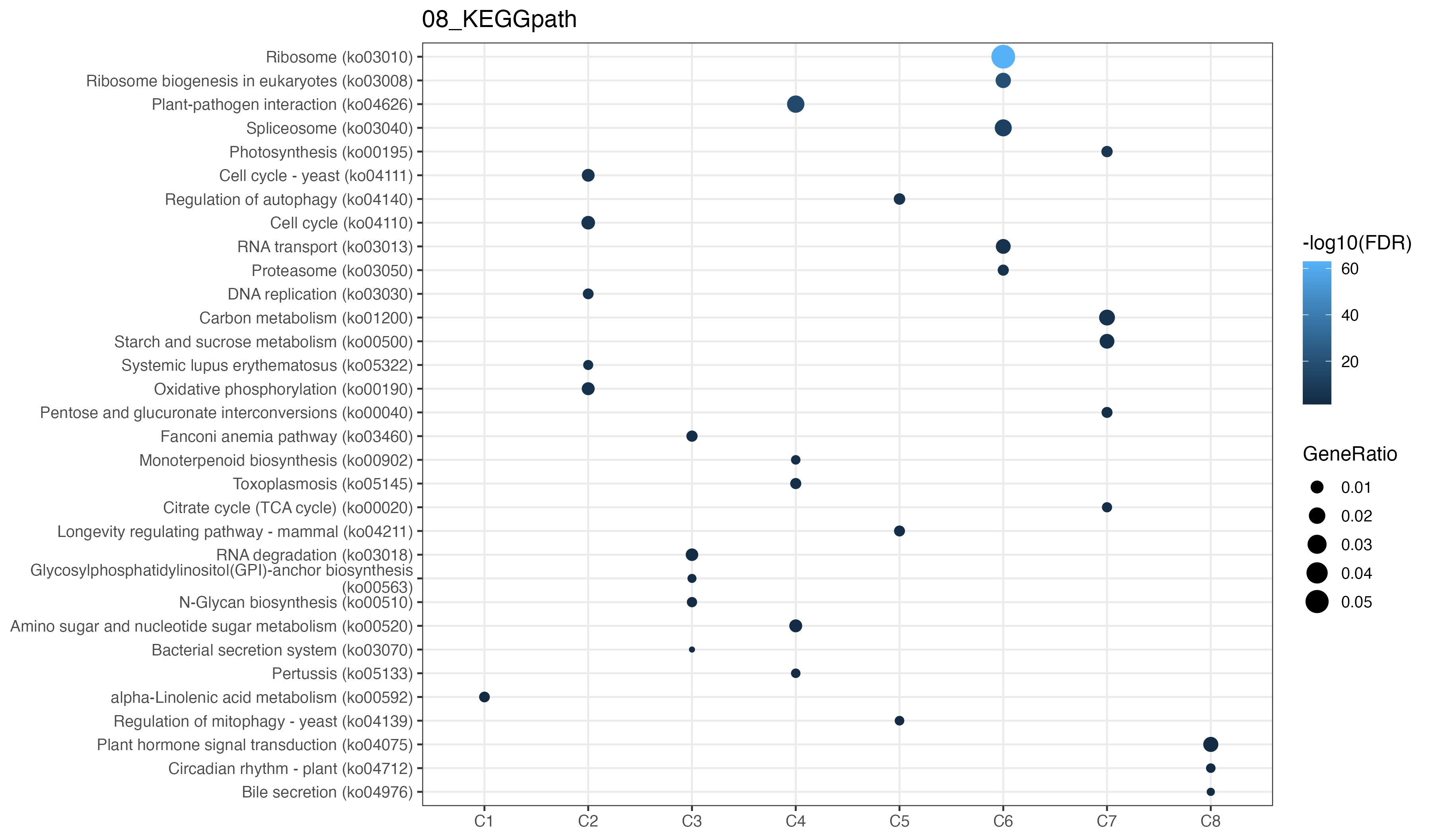


**Supplementary Figure 4. KEGG pathway enrichment for each trajectory cluster.** Pathway enrichment provides higher-order biological process context for each temporal module; dot size indicates gene ratio and dot colour indicates −log10(FDR).
